# A Novel and Effective Recyclable BiOCl/BiOBr Photocatalysis for Lignin Removal from Pre-Hydrolysis Liquor

**DOI:** 10.3390/nano11112836

**Published:** 2021-10-25

**Authors:** Shengyu Zhang, Jiachuan Chen, Qianqian Jia, Qimeng Jiang, Jiaqiang Yan, Guihua Yang

**Affiliations:** State Key Laboratory of Bio-Based Material and Green Papermaking/Key Laboratory of Pulp & Paper Science and Technology of Education Ministry of China, Qilu University of Technology, Jinan 250353, China; zsyv587@163.com (S.Z.); jqq2121@163.com (Q.J.); m17862958311@163.com (J.Y.)

**Keywords:** pre-hydrolysis liquor, BiOCl/BiOBr, lignin, photocatalysis

## Abstract

The presence of lignin hampers the utilization of hemicelluloses in the pre-hydrolysis liquor (PHL) from the kraft-based dissolving pulp production process. In this paper, a novel process for removing lignin from PHL was proposed by effectively recycling catalysts of BiOCl/BiOBr. During the whole process, BiOCl and BiOBr were not only adsorbents for removing lignin, but also photocatalysts for degrading lignin. The results showed that BiOCl and BiOBr treatments caused 36.3% and 33.9% lignin removal, respectively, at the optimized conditions, and the losses of hemicellulose-derived saccharides (HDS) were both 0.1%. The catalysts could be regenerated by simple photocatalytic treatment and obtain considerable CO and CO_2_. After 15 h of illumination, 49.9 μmol CO and 553.0 μmol CO_2_ were produced by BiOCl, and 38.7 μmol CO and 484.3 μmol CO_2_ were produced by BiOBr. Therefore, both BiOCl and BiOBr exhibit excellent adsorption and photocatalytic properties for lignin removal from pre-hydrolysis.

## 1. Introduction

The lignocellulosic biomass has a promising future and has been identified as a predictable, feasible and sustainable resource for value-added products [1]. The efficient separation and transformation of lignocellulosic components is an effective way to realize its comprehensive utilization, including the deep eutectic solvents (DESs) treatment for component isolation [2], lignin-based composite for photocatalytic degradation [3] and the production process of kraft-based dissolving pulp [4]. After the pretreating and cooking of raw materials, hemicellulose-based PHL, lignin-based black liquor and cellulose-based dissolving pulp were obtained independently [5]. The dissolving pulp is mainly used to prepare cellulose ether, acetate fiber, viscose fiber and other cellulose derivatives, which can be used in textiles and cigarette filters.

In the pulp mill, PHL is conventionally mixed with black liquor and burned for the recovery of chemicals and energy, which is unprofitable [6]. The hemicellulose-derived saccharides (HDS) in PHL can be utilized in the production of various value-added products, such as platform chemicals, biomaterials and biofuels [7,8]. In addition, xylo-oligosaccharide, which can improve the health of animal intestinal systems, is widely used in biochemical synthesis in the pharmaceutical and food industries [9]. The utilization of HDS is essential for the biorefinery concept, which can improve the comprehensive and efficient utilization of lignocellulosic biomass, increase revenue sources and reduce pollution load for enterprises. However, the presence of non-saccharide compounds in the PHL, especially lignin, hinders the separation and utilization of HDS. Therefore, the elimination of lignin is essential for the production of hemicellulose-based value-added products from PHL [10].

Many methods have been proposed for lignin removal from PHL which have been reported in the literature, including: (a) acidification [11,12,13]; (b) adsorption with lime [14,15], activated carbon (AC) [16,17] and ion exchange resin [18,19]; (c) flocculation with polymers [20,21]; (d) nanofiltration or microfiltration [22,23,24]; and (e) polymerization with laccase [25,26] and horseradish peroxidase [27,28]. All these processes had certain practical challenges, so they used to be combined for purifying the sugars of PHL [29,30]. Besides, most lignin removal methods were non-recyclable, which would result in increased costs and even pollution. Many studies reported that AC-treated PHL could be economically reused, such as in solvent extraction and thermal regeneration. However, solvent extraction has limited recovery efficiency, and thermal regeneration is similar to the production process of activated carbon; the effect depends on the type of AC, adsorption material and the choice of process [31,32]. Therefore, as there are some difficulties in recycling AC, a more recyclable process should be explored for removing lignin from PHL.

Photocatalysis is a green technology with important application prospects in energy and the environment [33]. Since the first report about the photocatalytic hydrogen production performance of TiO_2_ by Fujishima and Honda in 1972 [34], numerous efforts have been concentrated on developing excellent semiconductor photocatalysts [35]. A new type of photocatalytic material, BiOX (X = F, Cl, Br, I), has attracted much attention from researchers all over the world with its excellent optical adsorption, electrical properties and high photocatalytic activity [36,37]. The essence of a semiconductor photocatalytic reaction is that the photocatalytic material absorbs the external radiant light energy, produces electron–hole pairs, and then conducts a series of chemical reactions with the adsorbed material on the catalyst surface. A photocatalytic reaction is one of the interactions between light and matter which is the fusion of photoreaction and catalytic reaction. The photo-excited catalyst generates electron–hole pairs and further acts through electron holes to generate a redox reaction [38,39]. Photocatalysis was highly considered as a desirable and green strategy which played an indispensable role in many fields [40]. Photocatalytic applications of semiconductors also received attention in the pulp and paper industry. For example, photocatalytic technology significantly increased the value of black liquor from papermaking [41], so it is destined to have a potential utility as PHL.

Compared with AC, the regeneration of catalysts—or, in other words, photocatalytic reaction—is a much simpler process. Photocatalytic degradation of lignin has many advantages: reaction conditions, low cost, full light energy utilization, low energy consumption, no secondary pollution, simple equipment operation, etc. The products are only CO, CO_2_ and H_2_O, while CO can be used for fuels or mineralized into CO_2_ by continued illumination.

The objective of the present study is to investigate a new recyclable method for removing lignin from PHL. The hypothesis is that BiOCl/BiOBr can remove lignin in PHL by electrostatic adsorption, and the catalyst can also be recycled after the complete degradation of lignin by photocatalysis. The process of removing lignin by BiOCl/BiOBr can also be combined with other processes, as the catalysts are stable and will not alter the chemistry of the PHL after treatment. In addition, the optimization of BiOCl/BiOBr treatment conditions for PHL are investigated, and the feasibility of repeated experiments of BiOCl/BiOBr is especially evaluated.

## 2. Methods

### 2.1. Materials

Ethanol (99.7% *v*/*v*) and 2-methoxyethanol (99.5% *v*/*v*) were purchased from Tianjin Fuyu Fine Chemical Company, Tianjin, China. Bi(NO_3_)_3_·5H_2_O was purchased from Sinopharm Chemical Reagent Company, Shanghai, China. C_16_MIMCl and C_16_MIMBr imidazole-based ionic liquids were purchased from Shanghai Chengjie Chemical Co., Ltd., Shanghai, China. H_2_SO_4_ (98% *v*/*v*) was purchased from Tianjin Hengxing Company, Tianjin, China. These chemical reagents were all analytical grades. The water used in the experiments was deionized water.

#### 2.1.1. PHL Preparation

Fast-growing poplar wood chips were obtained from Shan Dong Sun Paper Industry Joint Stock Co., Ltd., Jining, China. The washed wood chips were balanced in moisture by airing under sunlight, then qualified wood chips were picked and stored in a plastic receptacle at room temperature. Pre-hydrolysis of 500 g oven-dried wood chips was conducted in a horizontal rotary pulp digester (rotary type autoclave No.2611, Kumgai Riki Kogyo Co., Ltd. (Tokyo, Japan) with wood to water at a ratio of 1:6 (*w*/*w*), heated from room temperature to 170 °C at a speed of approximately 2.5 °C/min, and kept for 60 min. After pre-hydrolysis, the digester was rapidly taken off and cooled down with running water. Large particles and impurities in the PHL were removed by gauze in advance, and the liquid was filtered through a 0.22 μm nylon membrane, then placed in an encapsulated vial and stored at 4 °C before analysis.

#### 2.1.2. Synthesis of BiOCl/BiOBr

At room temperature, 2 mmol Bi(NO_3_)_3_·5H_2_O and 3 mmol C_16_MIMCl were separately dissolved in 40 mL 2-methoxyethanol by stirring. Afterwards, the C_16_MIMCl solution was dropped into the Bi(NO_3_)_3_·5H_2_O solution slowly, then the mixture was continuously stirred for 30 min and decanted into a 100 mL Teflon-lined stainless steel autoclave. The autoclave was transferred into a 160 °C oven, heated and maintained for 1 h, and then cooled to room temperature naturally. The resulting BiOCl was washed several times with ethanol and water alternately, and dried at 60 °C. Meanwhile, the synthesis of BiOBr was under the same circumstances of the BiOCl synthesis, except replacing C_16_MIMCl with C_16_MIMBr.

#### 2.1.3. BiOCl/BiOBr Treatment of PHL

A certain amount of BiOCl/BiOBr (1.0 wt% to 12.0 wt%) was added to 20 g PHL in a glass beaker, and the mixture was stirred at 350 rpm at room temperature. In addition, the effect of elapsed time (1 min to 60 min) on lignin removal was optimized. After the adsorption process, the solution was filtered through a 0.22 μm nylon membrane; subsequently, the filtrate was stored under the same condition as the PHL storage, and the precipitate was dried at 60 °C before photocatalytic regeneration.

#### 2.1.4. Photocatalytic Degradation of Lignin

The regenerations of used BiOCl/BiOBr were conducted in a 100 mL airtight glass round-bottom flask. The used BiOCl/BiOBr after drying was suspended in 50 mL water before the experiment, the reaction mixture was stirred gently, and the reactor was kept at 30 °C by a cooling water circulation system. A 300 W xenon lamp (PLS-SXE300, Beijing Trusttech Co., Ltd., Beijing, China) was irradiated from the top of reactor. The gas in the reactor was extracted with a syringe every 3 h, measured by gas chromatograph (CEL-GC7900, Shimane, Japan), and assembled with a flame ionization detector (FID); ultrapure nitrogen was used as a carrier gas.

#### 2.1.5. Cycle Experiments of BiOCl/BiOBr

The repeat experiments were carried out as previously mentioned. In detail, after 9 h photocatalysis in closed reactor, the mixture was exposed to air for illumination of 12 h to ensure the complete degradation of organic matter on the surface of the catalyst. After photocatalysis, solids and liquids were separated by filtration. Regenerated catalyst was recycled after drying, and the filtrate was preserved for subsequent analysis. Adsorption experiments were carried out with different cycle times under 6.0 wt% dosage and 10 min adsorption time at room temperature, and the regenerated catalysts were repeat-treated with 20 g fresh PHL.

#### 2.1.6. Characterization of BiOCl/BiOBr

Characterizations of fresh BiOCl/BiOBr, used BiOCl/BiOBr and regenerated BiOCl/BiOBr were all investigated. The crystal phases were performed on a Bruker AXS D8 X-ray diffraction (XRD) with Cu Kα radiation. The morphologies were observed by scanning electron microscopy (SEM, Hitachi Regulus8220). The UV–vis diffuse reflectance spectra (DRS) were measured on a Shimadzu UV-2550 recording spectrometer with BaSO_4_-coated integration sphere. The Fourier-transform infrared (FT-IR) spectroscopy was carried out on an ALPHA Fourier-transform infrared spectrometer.

#### 2.1.7. Zeta Potentials and pH Values

A lignin suspension (1 g/L) and a solution with xylose, arabinose, galactose, glucose and mannose (all 1 g/L) were prepared in advance. The pH values of the former two liquids were measured along with PHL and water, then the lignin suspension, sugar solution and water were adjusted to the pH of PHL by acetic acid. The zeta potentials of these adjusted liquids were measured by using a Malvern Zetasizer Nano ZSP (Malvern, UK).

#### 2.1.8. Analysis of PHL

The monosaccharide concentration in the PHL was determined by using an ion chromatography system (ICS-5000 + DC, Thermo Scientific, Waltham, MA, USA). The ion chromatography system was equipped with an ED40 electrochemical detector (Au working electrode, Ag/AgCl reference electrode), a CarboPA100 analytical column (3 mm × 150 mm) and a CarboPac PA100 protection column (3 mm × 30 mm). For measuring the concentration of total saccharides in the PHL, an additional acid hydrolysis step was carried out on the samples under the conditions of 4% (*w*/*w*) H_2_SO_4_ and 121 °C for 1 h.

The lignin content in the PHL was determined by a UV/vis spectrophotometer (Agilent Technologies, Palo Alto, CA, USA) at 205 nm. The PHL was diluted a certain number of times with water as the blank group, and the absorbance value of the sample at 205 nm was measured by UV/vis spectrophotometer (the absorbance value was in the range of 0.2~0.7). Lignin content of the PHL was calculated by following Equation (1):

*B* = *A*·*D*/110 (1)

where *A*, *D* and *B* represent the absorbance, dilution ratio and lignin content (g/L), respectively, and 110 is the absorption coefficient, L/(g·cm^−1^).

The properties of the prepared PHL were as follows: HDS, 19.4 g/L; monosaccharides, 4.7 g/L; oligosaccharides, 14.7 g/L; dissolved lignin, 4.4 g/L. Varying concentrations of monosaccharide standard samples (arabinose, galactose, glucose, xylose and mannose) were prepared and detected separately by ion chromatography, and the standard curve was fitted according to the corresponding peak area. The PHL diluted to a certain concentration was detected by ion chromatography. The corresponding monosaccharide concentration was calculated through the peak area. The concentration of HDS was the sum of the five sugar contents.

## 3. Results and Discussion

### 3.1. A Novel Lignin Removal Method by Electrostatic Adsorption

**Figure 1:**
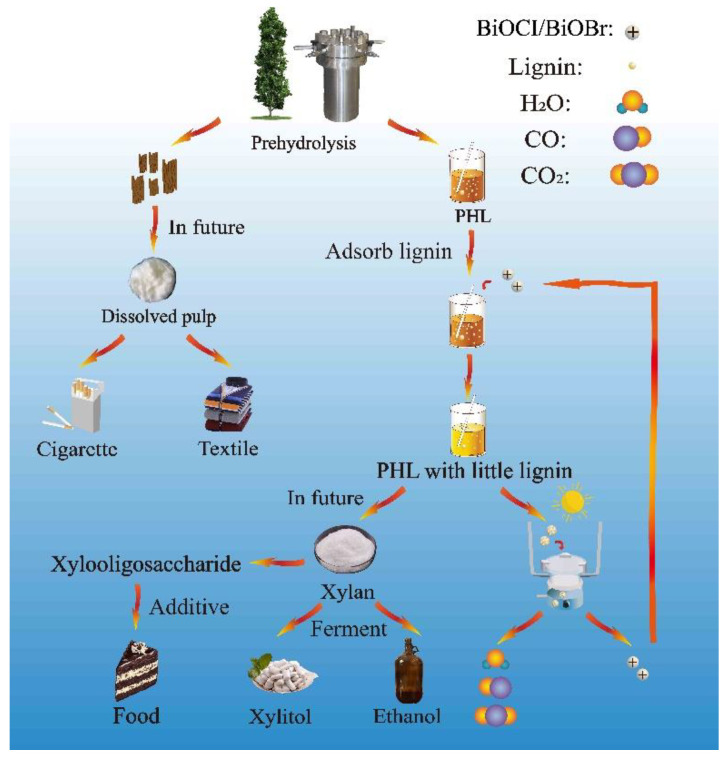
Process diagram for sustainable lignin removal by BiOCl/BiOBr in a kraft-based dissolving pulp production process.

The process of kraft-based dissolving pulp by pre-hydrolysis was considered as the most suitable production practice for biorefinery [5]. Figure 1 shows a proposed diagram for high-value utilization of lignocellulose by an environmental, recyclable and economical process. In the production process of dissolving pulp, hemicellulose and soluble small-molecular lignin can be dissolved and removed by hot water pre-hydrolysis. As discussed before, the removal of lignin is essential prior to the value-added utilization of HDS, and the BiOCl/BiOBr was employed to remove lignin from PHL. After treatment, the PHL contained less lignin, and the HDS could be conveniently recovered/utilized for value-added products by further purification. Lignin adsorbed on the surface of catalyst could be photocatalytically degraded into CO, CO_2_ and H_2_O, and the catalysts would be regenerated to be used for treating the PHL again.

### 3.2. Optimization of BiOCl/BiOBr Treatment Conditions

**Figure 2:**
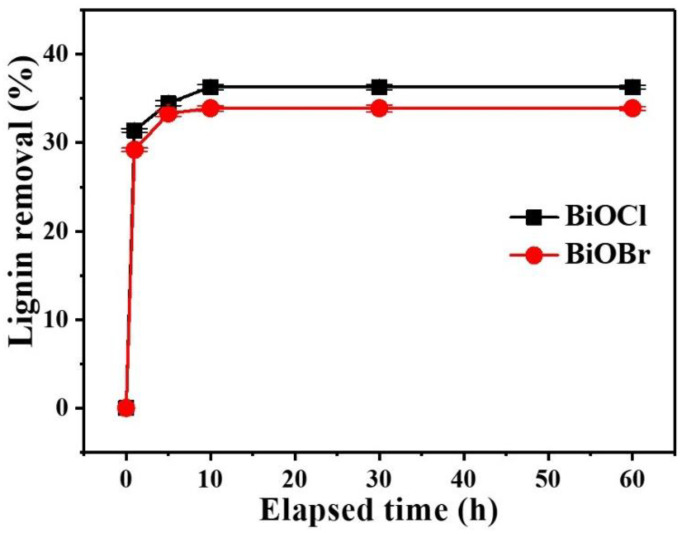
Effect of elapsed time on the lignin removal of PHL by BiOCl/BiOBr treatment.

Under the conditions of room temperature and adsorbent dosage 6.0 wt%, the effect of elapsed time on the removal of lignin in PHL by BiOCl/BiOBr is shown in Figure 2. Obviously, the adsorption experiment took place rapidly. The removal of lignin (relative to the lignin content in the pre-hydrolysis liquor) was 31.4% and 29.2% by BiOCl and BiOBr, respectively, at 1 min, then the adsorption equilibrium was achieved within 10 min, and 36.3% and 33.9% removals of lignin were achieved, respectively. This result indicates that the removal of lignin by BiOCl or BiOBr from PHL is a transitory process which is applicable for factories. Meanwhile, the processing time selected for the subsequent BiOCl/BiOBr treatments of the PHL was 10 min. As a comparison, the study investigated the effectiveness of wheat straw delignification, as well as the selectivity of the process, by using deep eutectic solvents. The highest lignin removal was observed for the ChCl and OX system (1:1, 57.9%), followed by the ChCl-lactic (1:10, 29.1%) and -malic (1:1, 21.6%) acid system [2]. The removed lignin could be synthesized for photoactive lignin/Bi_4_O_5_Br_2_/BiOBr bio-inorganic composites. The lignin-based composite decreased the dye concentration from 80 mg·L^−1^ to 12.3 mg·L^−1^ for RhB (85%) and from 80 mg·L^−1^ to 4.4 mg·L^−1^ for MB (95%). Therefore, the lignin as a main component of the composite was found to be an efficient and rapid biosorbent for nickel, lead, and cobalt ions [3].

**Figure 3:**
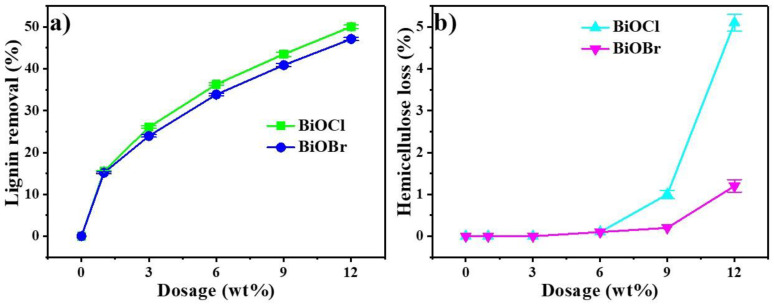
Effect of BiOCl/BiOBr dosage on the lignin removal (**a**) and HDS loss (**b**) of PHL.

In batch adsorption experiments, the removals of lignin and losses of HDS were measured at various adsorbent levels at 10 min and room temperature, and the results are shown in Figure 3. Figure 3a indicates that the removal of lignin was strongly affected by the dosage of BiOCl/BiOBr. Along with the increase in dosage from 1.0 wt% to 12.0 wt%, the removal of lignin increased from 15.5% to 50.1% by BiOCl, while the removal of lignin increased from 15.2% to 47.1% by BiOBr. The removals of lignin in the PHL by BiOCl and BiOBr were effective, and the loss of HDS should be minimized or even eliminated. Figure 3b shows the effect of BiOCl/BiOBr on the loss of HDS in the PHL. It can be seen that the losses of HDS were almost none at low dosage, and the losses of HDS in the PHL were both 0.1% at 6.0 wt% dosage of BiOCl and BiOBr, while the losses of HDS were 5.1% and 1.2% after 12.0 wt% dosage of BiOCl and BiOBr, respectively. The reason for this small portion of HDS lost was attributed to partial lignin adsorbed on BiOCl/BiOBr, which were covalently bonded to HDS [42]. The following experiments were conducted with 6.0 wt% dosage of BiOCl/BiOBr because the lignin removal was considerable and the HDS loss was negligible.

### 3.3. Affinity of Lignin to BiOCl/BiOBr

**Figure 4:**
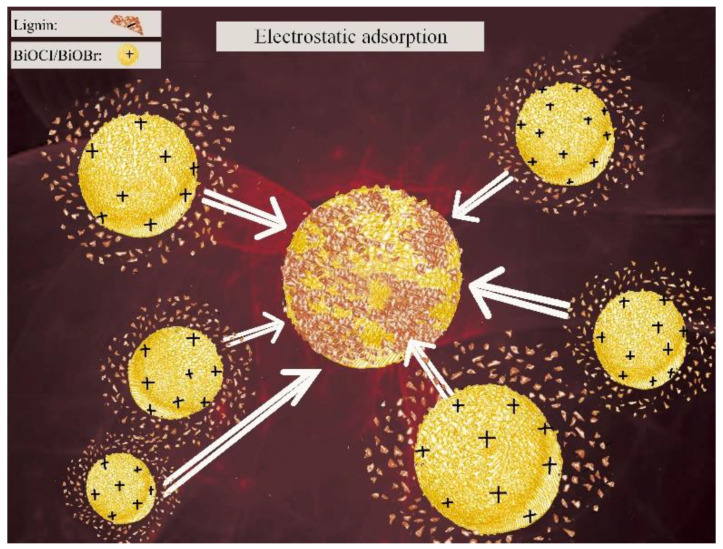
Schematic diagram of the electrostatic adsorption between BiOCl/BiOBr and lignin.

The strong adsorption of lignin was on account of its high molecular weight and being negatively charged on its surface, while the positive charges on the surface of BiOCl/BiOBr according to the determination of zeta potentials are shown in Table 1. A new lignin removal process was explored based on the zeta potentials of lignin suspension, sugar solution, BiOCl suspension and BiOBr suspension, as the resulting values were −21.2, −2.06, +8.7 and +2.8, respectively. It reveals that the lignin suspension was electronegative, the BiOCl/BiOBr suspension was electropositive and the sugar solution was close to neutral. Thus, the opposite charges on the lignin and the BiOCl/BiOBr surface should result in a strong electrostatic attraction between the two, as shown in Figure 4. The electronegativity of lignin was much higher than that of HDS, which caused the strong affinity of lignin and weak affinity of HDS to BiOCl/BiOBr. In addition, as seen from the zeta potentials, the electropositivity of BiOCl was stronger than BiOBr, which corresponded to the finding that the removal of lignin by BiOCl was better than BiOBr.

### 3.4. Characterization of BiOCl/BiOBr

**Figure 5:**
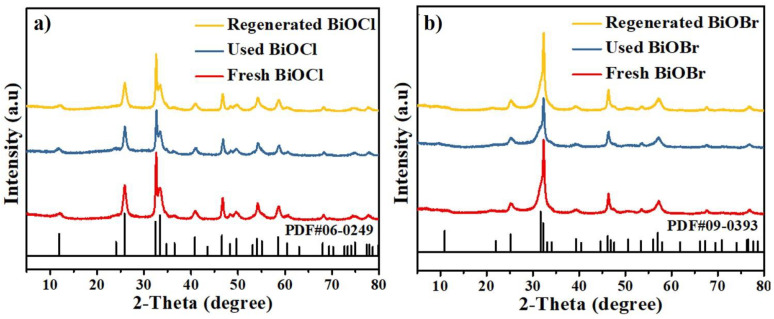
XRD images of BiOCl (**a**) and BiOBr (**b**).

The XRD spectra of fresh, used and regenerated BiOCl/BiOBr were displayed to investigate the crystalline structures, and the results are shown in Figure 5. As can be seen, all diffraction peaks of the products in Figure 5a could be matched to the tetragonal BiOCl (JCPDS no. 06-0249), and all diffraction peaks of the products in Figure 5b could be perfectly identified as tetragonal BiOBr (JCPDS no. 09-0393). Moreover, no additional diffraction peaks of impurities are detected, and this implies that synthesized catalysts had high purities and the structures of the catalyst remained after adsorption and photocatalysis. The used catalysts did not react with the organics in the PHL, and the generated catalysts certainly did not transform into Bi_2_O_3_ or Bi(OH)_3_. These are owing to the chemical stability of catalysts and having a tendency to complete the cycle of catalysts. In the photocatalytic experiment, reactive ⦁OH radicals could result in the degradation of lignin due to the scission β-O-4 bond. This process resulted in the generation of benzyl, alkoxy and alkyl free radicals, which took part in lignin depolymerisation reactions to form low molecular weight lignin fragments. The ⦁OH radicals could also directly attack the phenyl rings of the lignin to form catechol, resorcinol and hydroquinone, and the lignin could be completely mineralized to CO_2_.

**Figure 6:**
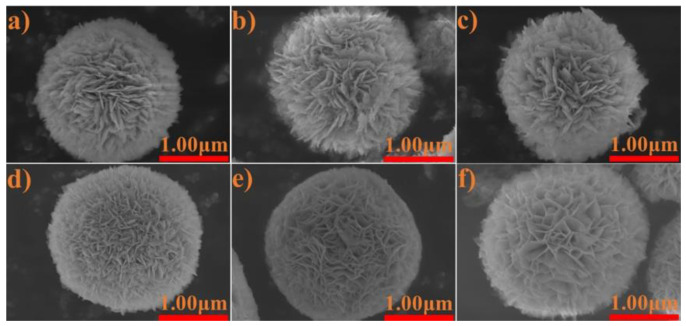
SEM images of fresh (**a**), used (**b**) and regenerated (**c**) BiOCl and fresh (**d**), used (**e**) and regenerated (**f**) BiOBr.

The SEM images of fresh, used and regenerated BiOCl/BiOBr are shown in Figure 6. It can be seen that both BiOCl and BiOBr were about 1–2 μm and exhibited hollow flower-like morphologies, which presented clusters of some interlaced nanosheets. The ultrathin nanosheet structures contributed to preferable specific surface area, which may be beneficial for the adsorption of lignin. Additionally, the used and regenerated catalysts held the same morphologies, which confirmed that adsorption and photocatalysis did not change the structures of catalysts; this demonstrated the feasibility of the cycle of BiOCl/BiOBr by photocatalysis once more.

**Figure 7:**
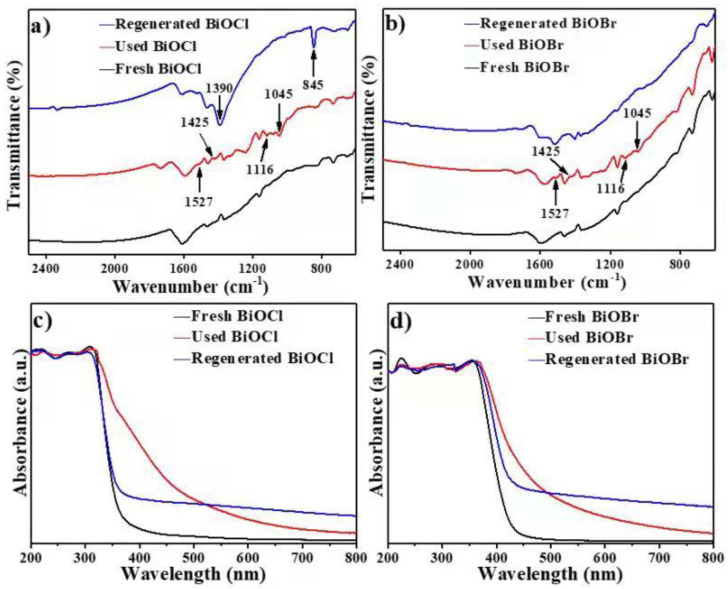
FT-IR spectrum of BiOCl (**a**) and BiOBr (**b**); UV-vis absorption spectra of BiOCl (**c**) and BiOBr (**d**).

As shown in Figure 7a,b, the major features of FT-IR spectra for these three samples of BiOCl were also similar, except that the four new peaks appeared after the lignin adsorption step was performed, with a similar result in BiOBr. The peaks at 1045 cm^−1^ and 1116 cm^−1^ could be ascribed to aromatic C-H in-plane deformation in the syringyl ring; the peaks at 1425 cm^−1^ corresponded to the C-H in-plane deformation with aromatic ring stretching; and the peaks at 1527 cm^−1^ could be assigned to aromatic skeletal vibration [43]. It was confirmed from FT-IR spectra that a portion of lignin was adsorbed onto BiOCl and BiOBr particles in the process of adsorption treatment. After the complete photocatalytic conversion of the adsorbed lignin, the characteristic FT-IR peaks of lignin disappeared. During the photocatalysis of lignin by BiOCl, some intermediate CO_3_^2^^−^ ion degradation of lignin was produced. The fundamental vibrations arose from the carbonate CO_3_^2^^−^ ion and were assigned to the asymmetric stretch (v_3_) at 1390 cm^−1^ and the out-of-plane bending (v_2_) vibration at 845 cm^−1^. This phenomenon was considered to be among the prominent absorption features within carbonate spectra. Figure 7c,d each display the UV–vis DRS spectra of fresh, used and regenerated BiOCl and BiOBr. Used catalysts exhibited obvious red-shift compared to fresh catalysts, which corresponded to the changes of samples’ colours—the fresh catalysts were both white, then changed into yellow after the adsorption of lignin. The visible light of absorption of both regenerated catalysts increased significantly, which may be attributed to the generation of oxygen vacancy after photocatalysis, as the regenerated catalysts both changed into dark colour [44].

### 3.5. Photocatalytic Degradation of Lignin

**Figure 8:**
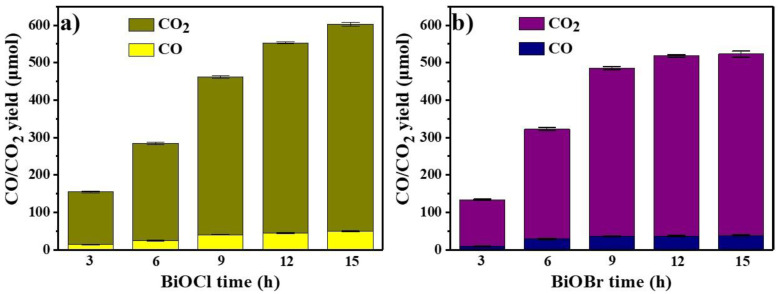
Effect of illumination time on the yield of CO/CO_2_ produced by photocatalysis of lignin by BiOCl (**a**) and BiOBr (**b**).

Lignin on surface of BiOCl/BiOBr degraded into CO, CO_2_ and H_2_O, etc. by photocatalysis, and the yields of gases (CO and CO_2_) by BiOCl and BiOBr are shown in the Figure 8a,b, respectively. These illustrate the photocatalytic degradation mechanism of the lignin adsorbed on the surface of catalysts. CO and CO_2_ were significantly increased with the extension of illumination time, which was due to the further degradation of lignin. The reaction rates of lignin degradation by BiOCl and BiOBr were both significant within 9 h, and further increase in the illumination time had ordinary effects on the yields of CO/CO_2_, which may be caused by the consumption of lignin and limited O_2_ in the hermetic bottle. By 15 h photocatalysis, 50.1 μmoL CO and 556.0 μmoL CO_2_ were obtained by BiOCl, while 38.9 μmoL CO and 487.3 μmoL CO_2_ were obtained when using BiOBr.

### 3.6. Cycle Performance of BiOCl/BiOBr

**Figure 9:**
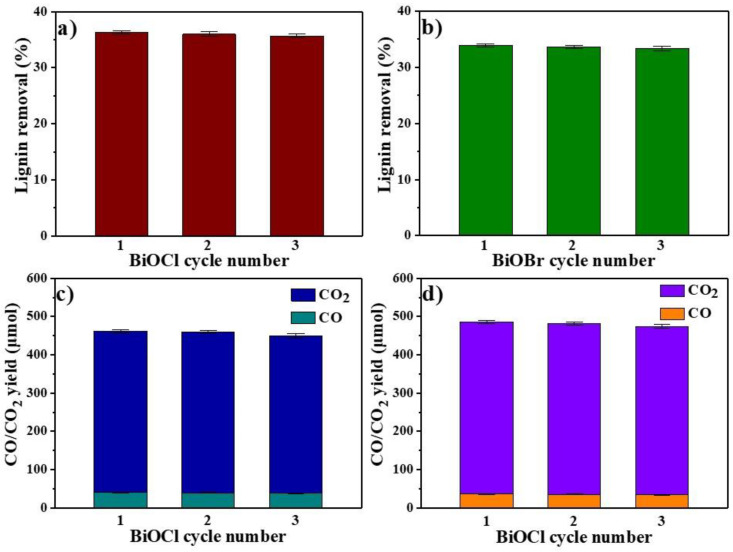
Lignin removals from PHL by cycled (**a**) BiOCl and (**b**) BiOBr; yield of CO/CO_2_ produced by photocatalysis of lignin by cycled BiOCl (**c**) and BiOBr (**d**).

The used BiOCl and BiOBr were regenerated by photocatalysis, then the adsorbents were used to perform adsorb experiments with PHL under the same circumstances after degradation of the lignin adsorbed on their surface. The cycle experiment was carried out under 6.0 wt% dosage BiOCl and BiOBr for 10 min; as shown in Figure 9a,b, the removals of lignin by the regenerated adsorbent maintained well. The lignin removal rate dropped to 35.7% and 33.3% at the third time of BiOCl and BiOBr, respectively, which might be caused by the loss of adsorbent during filtration and drying in the experiments. The tiny decrease was unavoidable, so the cycle of BiOCl and BiOBr remained a remarkable adsorptive property.

The yields of CO and CO_2_ in three cycles by BiOCl and BiOBr are shown in Figure 9c,d. As can be seen, the yields of CO and CO_2_ decreased a little upon repeat experiments because of the loss of catalyst during experiments. The finding was in agreement with the reduction in the lignin removal. The decrease in lignin removal was also an important reason, as the lignin adsorbed on the catalysts tailed off. At the first experiment, 40.4 μmoL CO and 424.8 μmoL CO_2_ were obtained by 9 h BiOCl photocatalysis, which then dropped to 39.1 μmoL CO and 410.7 μmoL CO_2_ at the third cycle. Furthermore, 36.3 μmoL CO and 452.8 μmoL CO_2_ were obtained by 9 h BiOBr photocatalysis the first time, which then dropped to 35.0 μmoL CO and 441.9 μmoL CO_2_ after three cycles. This analysis shows that BiOCl and BiOBr regenerated by photocatalysis can be reused effectively, which is beneficial for large-scale implementation.

## 4. Conclusions

In summary, BiOCl/BiOBr treatment for lignin removal from PHL by electrostatic adsorption was very rapid, and equilibrium could be achieved within 10 min. Under the room temperature and 6.0 wt% dosage of BiOCl and BiOBr, 36.3% and 33.9% lignin were removed, respectively, and the losses of HDS were both 0.1%. The adsorbed lignin could transform into CO, CO_2_ and H_2_O, etc. By 15 h illumination, 50.1 μmoL CO and 556.0 μmoL CO_2_ were obtained by BiOCl, and 38.9 μmoL CO and 487.3 μmoL CO_2_ were obtained by BiOBr. The catalysts were regenerated by the photocatalysis, and both BiOCl and BiOBr can be recycled and keep extreme adsorption and photocatalytic properties. Compared with traditional treatments of PHL, this novel process is more economical and environmentally friendly. Therefore, the catalysts have potential application for lignin removal from pre-hydrolysis liquor.

## Figures and Tables

**Table 1:** Initial pH values and zeta potentials of adjusted liquids.

	Initial pH	Zeta Potential (mV)
PHL	3.48	−2.84
DI water	5.50	−1.02
1 g/L lignin suspension	7.31	−21.2
1 g/L sugar solution	5.42	−2.06

Note: Zeta potentials of BiOCl and BiOBr suspension were +8.7 and +2.8.

## Data Availability

This manuscript comprises an original, unpublished material, which is not under consideration for publication elsewhere, and all authors have read and approved the text and consent to its publication. All experimental data are accurate and reliable.
